# Long-Term Exposure to Ambient Fine Particulate Matter and Renal Function in Older Men: The Veterans Administration Normative Aging Study

**DOI:** 10.1289/ehp.1510269

**Published:** 2016-03-08

**Authors:** Amar J. Mehta, Antonella Zanobetti, Marie-Abele C. Bind, Itai Kloog, Petros Koutrakis, David Sparrow, Pantel S. Vokonas, Joel D. Schwartz

**Affiliations:** 1 Exposure, Epidemiology and Risk Program, Department of Environmental Health, Harvard T.H. Chan School of Public Health, Boston, Massachusetts, USA; 2 Department of Geography and Environmental Development, Ben-Gurion University of the Negev, Beer Sheva, Israel; 3 VA Normative Aging Study, Veterans Affairs Boston Healthcare System, Boston, Massachusetts, USA; 4 Channing Division of Network Medicine, Brigham and Women’s Hospital, Harvard Medical School, Boston, Massachusetts, USA; 5 Department of Medicine, Boston University School of Medicine, Boston, Massachusetts, USA

## Abstract

**Background:**

It is unknown if ambient fine particulate matter (PM2.5) is associated with lower renal function, a cardiovascular risk factor.

**Objective:**

We investigated whether long-term PM2.5 exposure was associated with estimated glomerular filtration rate (eGFR) in a cohort of older men living in the Boston Metropolitan area.

**Methods:**

This longitudinal analysis included 669 participants from the Veterans Administration Normative Aging Study with up to four visits between 2000 and 2011 (n = 1,715 visits). Serum creatinine was measured at each visit, and eGFR was calculated according to the Chronic Kidney Disease Epidemiology Collaboration equation. One-year exposure to PM2.5 prior to each visit was assessed using a validated spatiotemporal model that utilized satellite remote-sensing aerosol optical depth data. eGFR was modeled in a time-varying linear mixed-effects regression model as a continuous function of 1-year PM2.5, adjusting for important covariates.

**Results:**

One-year PM2.5 exposure was associated with lower eGFRs; a 2.1-μg/m3 interquartile range higher 1-year PM2.5 was associated with a 1.87 mL/min/1.73 m2 lower eGFR [95% confidence interval (CI): –2.99, –0.76]. A 2.1 μg/m3-higher 1-year PM2.5 was also associated with an additional annual decrease in eGFR of 0.60 mL/min/1.73 m2 per year (95% CI: –0.79, –0.40).

**Conclusions:**

In this longitudinal sample of older men, the findings supported the hypothesis that long-term PM2.5 exposure negatively affects renal function and increases renal function decline.

**Citation:**

Mehta AJ, Zanobetti A, Bind MC, Kloog I, Koutrakis P, Sparrow D, Vokonas PS, Schwartz JD. 2016. Long-term exposure to ambient fine particulate matter and renal function in older men: the VA Normative Aging Study. Environ Health Perspect 124:1353–1360; http://dx.doi.org/10.1289/ehp.1510269

## Introduction

Chronic exposure to ambient fine particulate matter (PM_2.5_) is a well-known risk factor for cardiovascular-related morbidity and mortality (Brook et al. 2010). Although the underlying mechanisms have not been fully elucidated, there is evidence suggesting that pathways at the molecular level, including inflammation (Ostro et al. 2014; Rückerl et al. 2014) and oxidative stress (Sørensen et al. 2003), and at the function level, including arterial blood pressure (BP) (Fuks et al. 2014; Liang et al. 2014) and vascular/endothelial function (Krishnan et al. 2012; Wilker et al. 2014), may have a role in PM_2.5_-related cardiovascular morbidity and mortality.

It is also hypothesized that renal function impairment may be a mediating factor of the cardiovascular effects of long-term PM_2.5_ exposure because the kidney is a vascularized organ susceptible to large-vessel atherosclerotic disease and microvascular dysfunction (Lue et al. 2013). Impaired renal function, as determined from the estimated glomerular filtration rate (eGFR), is also associated with cardiovascular events and mortality (Fox et al. 2012; Go et al. 2004; Sarnak et al. 2003). There is limited experimental evidence suggesting that particle exposure affects the kidney; *in vivo* studies in rats have shown controlled exposure to urban or diesel exhaust particles to be associated with increased cytokine expression in the kidney (Thomson et al. 2013) and to aggravate acute renal failure (Nemmar et al. 2010). A recent cross-sectional study of stroke patients living in the Boston metropolitan area found that living near a major roadway was associated with reduced eGFR (Lue et al. 2013). To our knowledge, no longitudinal population study has directly evaluated whether long-term PM_2.5_ exposure is associated with reduced renal function or with increased age-related decline in renal function.

We investigated whether 1-year averaged exposure to PM_2.5_ was associated with reduced renal function and with renal function decline over time in an ongoing prospective cohort study of older men living in the Boston metropolitan area. This study takes advantage of a longitudinal sample with serum creatinine measured at multiple visits, where renal function was characterized by creatinine-based eGFR as defined by the Chronic Kidney Disease Epidemiology Collaboration (CKD-EPI) (Levey et al. 2009), as well as a validated, spatially and temporally resolved prediction model of annual outdoor exposure to PM_2.5_ at each participant’s home address (Kloog et al. 2011, 2014). We also investigated whether the association between PM_2.5_ and eGFR was mediated via a change in BP.

## Methods

Participants included in this analysis were enrolled in the Veterans Administration (VA) Normative Aging Study, an ongoing longitudinal study of aging established by the VA in 1963, details of which have been published previously (Bell et al. 1972). Briefly, the VA Normative Aging Study is a closed cohort of 2,280 male volunteers from the greater Boston area (Massachusetts), 21–80 years of age at study entry, who enrolled after an initial health screening determined that they were free of known chronic medical conditions. The study population is predominantly White; < 2% of all participants are Black. Participants have been reevaluated every 3–5 years with detailed, onsite physical examinations and questionnaires. Visits took place in the morning, after an overnight fast and smoking abstinence. The present study was approved by the human research committees of the Harvard T.H. Chan School of Public Health and the Veterans Affairs Boston Healthcare System, and written informed consent was obtained from subjects before participation.

**Figure 1:**
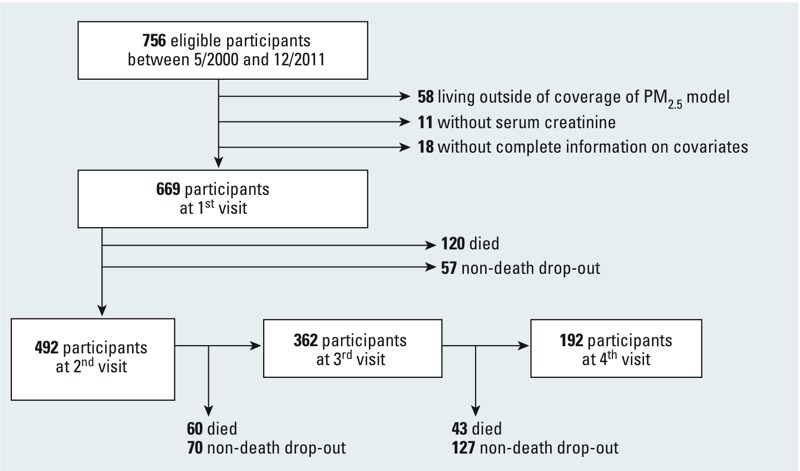
Flowchart describing inclusion of participants in analysis. PM_2.5_, fine particulate matter ≤ 2.5 μm in diameter.

Eligibility for this study required continued participation between May 2000 and December 2011, the period for which satellite-derived aerosol optical depth (AOD) measurements were available, and 756 participants had ≥ 1 scheduled visit during this time period (Figure 1). Of the 756 participants, we excluded 58 (7.7%) for not continuing to live within the coverage area of the prediction model, 11 (1.5%) for not having serum creatinine measured at any visit, and 18 (2.3%) for having incomplete information on covariates of interest. Thus, a total of 669 participants were included in this longitudinal repeated-measures analysis and presented for a total of 1,715 visits during the study period.

### Exposure Assessment

We assessed long-term PM_2.5_ exposure using satellite-based spatiotemporal models that predicted the daily ambient concentration of PM_2.5_ at each participant’s residence. We applied our recently published (Kloog et al. 2014) high-resolution 1 × 1 km exposure model for the years for which we had prediction data (2003–2011), and we applied a slightly lower-resolution 10 × 10 km model (Kloog et al. 2011) for the years for which new model predictions were unavailable (2000–2002). The 1 × 1 km exposure assessment is based on a new multi-angle implementation to atmospheric correction (MAIAC) algorithm developed by the National Aeronautics and Space Administration (NASA) (Lyapustin et al. 2011), whereas the 10 × 10 km exposure assessment is based on the older NASA moderate resolution imaging spectroradiometer (MODIS) algorithm. The MAIAC algorithm provides finer-resolution AOD data than the MODIS algorithm, which allowed us to use a high-resolution 1 × 1 km model versus the MODIS 10 × 10 km AOD data.

The 1 × 1 km and the 10 × 10 km models use similar methodology. We used a mixed-effects model approach by regressing daily PM_2.5_ mass concentrations (μg/m^3^) from the U.S. Environmental Protection Agency (EPA) Air Quality System and Interagency Monitoring of Protected Visual Environments network against the AOD, spatial predictors, and temporal predictors. For days when AOD data were not available for some grid cells, we regressed the predicted PM_2.5_ for each grid cell against the PM_2.5_ at the nearest monitor within 60 km of the cell and a thin-plate spline term of latitude and longitude. We used this model to fill in the missing grid cell–days. The mean out-of-sample *R* ^2^ values from 10-fold cross-validation were 0.88 and 0.83 for the 1 × 1 km and the 10 × 10 km models, respectively, indicating excellent prediction ability. Additional details are available in our previously published papers (Kloog et al. 2011, 2014). For each participant, we calculated address-specific exposure of 1-year averaged PM_2.5_ concentrations prior to examination visit.

### Outcome Assessment

Serum samples were drawn after overnight fasting, and serum creatinine concentration (Scr, mg/dL) was determined using a computerized automatic analyzer [Technicon SAM models (Technicon Corp., Tarrytown, NY) from 1979 to 1993; Boehringer Mannheim/Hitachi 747 analyzer (Boehringer-Mannheim Corp, Indianapolis, IN) from 1993 onward] at each examination. The analyzer measured creatinine based on the Jaffe procedure (Jaffe 1886) and showed excellent reproducibility. This method of analysis had an intraassay coefficient of variation (CV) of 1.3% at 1.2 mg/dL and an interassay CV of 3.3% at 1.1 mg/dL. We calculated eGFR at each visit using the CKD-EPI equation (eGFR = 141 × min(Scr/0.9, 1)^–0.411^ × max(Scr/0.9, 1)^–1.209^ × 0.993^Age^ × 1.159[if Black]) (Levey et al. 2009). The calculated intraclass correlation coefficient of 0.7 for eGFR indicated a high degree of stability over time.

### Ascertainment of Covariates

At each examination, participants underwent physical examination, their medical history was updated, and biomarkers from serum samples were measured, including fasting glucose and serum cholesterol. Serum cholesterol was assayed over the course of the study period using standard enzymatic methods and reagents (SCALVO Diagnostics, Wayne, NJ). Weight and height were measured with participants wearing only socks and underwear, from which body mass index (BMI; kg/m^2^) was calculated. Systolic blood pressure (SBP) and diastolic blood pressure (DBP) were measured with a standard cuff once in each arm while the subject was seated; the mean of the right and left arm values was used for analysis. At each visit, participants were also asked to bring their current medical prescriptions; antihypertensive medication use included any of the following classes: angiotensin converting enzyme inhibitors (ACEIs), diuretics, beta blockers, angiotensin receptor blockers (ARBs), calcium channel blockers, and alpha blockers. As a proxy measure of childhood socioeconomic status, parental homeownership during the participant’s childhood was ascertained by questionnaire at the baseline survey. As a proxy measure of adult socioeconomic status, maximum years of education was ascertained by questionnaire at the early visits. Year 2000 census data were obtained from the U.S. Bureau of the Census Summary File (III) (U.S. Census Bureau 2002) at the census tract level. For this analysis, we used the proportion of the population within a census tract living below the poverty level as an area-based measure of socioeconomic status.

### Statistical Analysis

All statistical analyses were performed using SAS v.9.3 software (SAS Institute Inc., Cary, NC). We used time-varying linear mixed-effects regression models with random intercepts accounting for the correlation of repeated measures (Fitzmaurice et al. 2004) to model eGFR (mL/min/1.73 m^2^) as a continuous function of 1-year PM_2.5_ scaled per interquartile range (IQR) and adjusting for age at first visit and time since first visit (years). To evaluate whether 1-year PM_2.5_ was associated with annual change in eGFR, we added an interaction between 1-year PM_2.5_ and time since first visit. We additionally adjusted for the following covariates ascertained at each visit including BMI (kg/m^2^), total cholesterol (mg/dL), coronary heart disease (no as reference), diabetes (physician-diagnosed or fasting blood glucose ≥ 126 mg/dL, no as reference), ARB use (no as reference), ACEI use (no as reference), other antihypertensive medication use (no as reference), years of education (< 12 years; 12 years; 13–15 years; ≥ 16 years as reference), percent of population in census tract living below poverty level, parental homeownership (no as reference), smoking status (current; former; never as reference), cumulative pack-years smoked, and daily alcohol intake (≥ 2 drinks/day; < 2 drinks/day as reference). The normality of the residuals in the linear mixed-effects model of eGFR and 1-year PM_2.5_ was determined in a preliminary analysis. We also evaluated the following participant characteristics as potential effect modifiers of the association between 1-year PM_2.5_ exposure and eGFR by constructing multiplicative interaction terms between each potential modifier and 1-year PM_2.5_, evaluating each separately in linear mixed-effects regression models: smoking status (current or former, never), high BP (SBP ≥ 140 mmHg and/or DBP ≥ 90 mmHg), diabetes mellitus, obesity (BMI ≥ 30 kg/m^2^), coronary heart disease, and use of ARB and ACEI medications. All tests for main associations and interactions for which *p* < 0.05 were considered statistically significant.

### Secondary Analyses

Because approximately 26% of the study participants did not have a repeat visit in this analysis, we conducted a baseline cross-sectional analysis of eGFR using multiple linear regression, excluding the available repeat visits from participants. We additionally adjusted for distance to nearest major roadway, with major roadway defined as those roads having U.S. Census Feature Class Code A1 (primary highway with limited access) or A2 (primary road without limited access), as in previous studies (Auchincloss et al. 2008; Lue et al. 2013). We also adjusted for eGFR measured at first visit to evaluate its influence on the interaction between 1-year PM_2.5_ and time since first visit. A total of 152 participants (~23%) began the study period with eGFR ≤ 60 mL/min/1.73 m^2^, presenting for a total of 331 visits during the study period. We also examined if the association between 1-year PM_2.5_ and eGFR was modified by the presence of impaired renal function (eGFR ≤ 60 mL/min/1.73 m^2^) at the first visit by including an interaction term in the model.

During the study period, 223 deaths (33%) and 254 nondeath dropouts (38%) occurred (Figure 1). To account for potential selection bias by age-dependent censoring from death and from nondeath dropout before the next visit, we applied stabilized inverse probability weights analogous to methods previously applied in the NAS cohort (Power et al. 2013). Estimation of stabilized inverse probability weights for each censoring mechanism required two logistic regression models, which predicted the probability of not being censored (death, nondeath dropout) at the next visit. The first logistic regression modeled death (nondeath dropout) before the next visit as a function of 1-year PM_2.5_. The second logistic regression modeled death (nondeath dropout) as a function of 1-year PM_2.5_, eGFR, age, and education, adjusting for an array of clinical and sociodemographic factors that might be associated with death and/or morbidity. Given the large number of potential predictors of censoring available in this study, we used forward stepwise selection to choose variables for inclusion in these logistic regression models, setting the maximum number of variables chosen to the number of participants who died or dropped out before each visit divided by 10, while forcing in 1-year PM_2.5_, renal function, age, and education-related variables. Additional variables in the models to predict censoring because of death before the next visit included the interaction between age and years of education, exclusion from pulmonary function testing for health reasons, physician’s diagnosis of emphysema, coronary heart disease, white blood cell count, smoking status, and pack-years smoked. Additional variables in the models to predict censoring because of nondeath dropout before the next visit included the interaction between age and years of education, parental homeownership, calendar year of visit, systolic blood pressure, ACEI medication use, calcium channel blocker use, statin medication use, cholesterol, serum hematocrit, fasting blood glucose, waist circumference, white blood cell count, diabetes mellitus, and current cancer diagnosis. Subsequently, we estimated stabilized inverse probability weights for censoring (death, nondeath dropout) before the next visit by taking the ratio of the probabilities estimated from the first and second logistic regression models. All weighted models met the necessary condition for correct model specification such that the mean value for the stabilized weights was approximately equal to 1 (Hernán et al. 2000). To compute a summary weight that combined the censoring by death and nondeath dropout, we simply multiplied the inverse probability weights for censoring by death and by nondeath dropout together.

Considering the evidence supporting an association between long-term PM_2.5_ and BP (Fuks et al. 2014; Liang et al. 2014), and the association between BP and eGFR decline (Young et al. 2002), we hypothesized that the association between long-term PM_2.5_ and reduced renal function is mediated through an increase in BP. Previously, we found that 1-year PM_2.5_ was associated with higher DBP in the NAS cohort (unpublished). Using methods previously developed and applied in the NAS cohort (Bind et al. 2014, 2016), we conducted a mediation analysis and calculated the estimated mediated effect of 1-year PM_2.5_ on eGFR through an increase in DBP. We simultaneously fit two time-varying linear mixed-effects models:

M_ij_ = (γ_0_ + u_i_) + γ_1_X_ij_ + Σ_k_ γ_2k_ C_kij_ + ε_ij_ with ε_ij_ ~N(0, σ^2^) and u_i_ ~N(0,σ_u_^2^), [1]

Y_ij_ = (β_0_ + g_i_) + β_1_ X_ij_ + β_2_M_ij_ + Σ_k_ β_3k_ C_kij_ + Σ_k_ β_4k_ + η_ij_ with η_ij_ ~ N(0, σ^2^), and g_i_ ~ N(0,σ_g_^2^), [2]

where i, j, and k are defined as individuals, visits, and covariates, respectively; M, X, and Y represent the DBP, 1-year PM_2.5_, and eGFR, respectively; C represents the set of covariates for which we adjusted, and g represents the random intercept for M. The estimated mediated effect is given by the product formula γ_1_β_2_. The delta method allowed us to approximate the variance of the estimated mediated effect by Var(β_2_) γ_1_^2^ + 2 Cov(γ_1_, β_2_) γ_1_ β_2_ + Var(γ_1_)β_2_^2^. The mediation formulae we have derived are valid if four identification assumptions are met, if there is no time-varying confounding with respect to the 1-year PM_2.5_ and DBP (which can be achieved in situations with exogenous exposure and mediator depending only on recent values of the exposure and confounders). The four identification assumptions include *a*) no unmeasured 1-year PM_2.5_–eGFR confounding at time j given the covariates and random effects, *b*) no unmeasured DBP–eGFR confounding, *c*) no unmeasured 1-year PM_2.5_–DBP confounding, and *d*) no DBP–eGFR confounder affected by 1-year PM_2.5_. Because the measurements of the mediator and the outcome are taken at the same time (cross-sectionally), it is implied that the mediator does not necessarily precede the measurement of the outcome, and we cannot rule out that the outcome could be the mediator and the mediator the outcome.

## Results

The characteristics of the participants at the first visit and over all visits are summarized in Table 1. The mean age of the participants was 73.5 years, and the majority of the participants were former smokers and used anti-hypertensive medication at the first visit. Compared with participants at the first visit, the mean eGFR was lower in participants over all visits, whereas the prevalences of coronary heart disease, hypertension, and of using anti-hypertensive medications were higher.

**Table 1:** Characteristics of participants at first visit and over all visits [mean ± SD or *n* (%)].

Characteristics	First visit *n* = 669	All visits *n *= 1,715
Age, years	73.5 ± 6.8	75.9 ± 6.8
eGFR (CKD-EPI), mL/min/1.73 m^2^	71.0 ± 15.7	68.0 ± 16.2
Body mass index, kg/m^2^	28.2 ± 4.1	27.9 ± 4.2
Serum creatinine, mg/dL	1.1 ± 0.5	1.1 ± 0.5
Total cholesterol, mg/dL	195.5 ± 38.5	183.8 ± 38.3
Years of education	15.0 ± 3.0	15.1 ± 2.9
Percent below poverty level (census tract 1999)	6.1 ± 5.6	6.0 ± 5.4
PM_2.5_ (1-year average), μg/m^3^	11.4 ± 1.0	10.5 ± 1.4
Smoking status
Current	31 (5)	55 (3)
Former	450 (67)	1,160 (68)
Never	188 (28)	500 (29)
Daily alcohol intake
≥ 2 drinks/day	124 (19)	315 (18)
< 2 drinks/day	545 (81)	1,400 (82)
Parents owned home	250 (37)	643 (37)
Obese (≥ 30 kg/m^2^)	175 (26)	438 (26)
Diabetes	117 (17)	332 (19)
Coronary heart disease	204 (30)	597 (35)
Hypertension diagnosis	474 (71)	1,298 (76)
High blood pressure (systolic blood pressure ≥ 140 mmHg or diastolic blood pressure ≥ 90)	186 (28)	402 (23)
Angiotensin receptor blocker medication use	31 (5)	144 (8)
Angiotensin converting enzyme inhibitor medication use	188 (28)	625 (36)
Other antihypertensive medication use (calcium channel blockers, beta blockers, or alpha blockers)	378 (57)	1,097 (64)
Abbreviations: CKD-EPI: Chronic Kidney Disease Epidemiology Collaboration equation; eGFR, estimated glomerular filtration rate; PM_2.5_, particulate matter ≤ 2.5 μm in diameter.		

The mean 1-year average PM_2.5_ concentrations at participant addresses at the first visit was 11.4 μg/m^3^ (Table 1). This estimate was lower in participant addresses over all visits; the mean was 10.5 μg/m^3^. All yearly averages from 2000 to 2011 were below the U.S. annual fine particle standard, which was recently revised downward from 15 μg/m^3^ to 12 μg/m^3^ in 2013 (U.S. EPA 2013), and the mean and median 1-year PM_2.5_ concentrations closely approximated each other (Table 2). In all person-visits by calendar year, the mean yearly average PM_2.5_ concentrations increased from 10.5 μg/m^3^ in 2000 to their highest level of 11.8 μg/m^3^ in 2002 and later decreased to levels < 9.0 μg/m^3^ in 2010 and 2011.

**Table 2:** Distribution of 1-year PM_2.5_ concentrations over all visits by calendar year.

Year	Visits (*n*)	Mean (SD)	Median (25th percentile, 75th percentile)
2000	141	10.5 (0.8)	10.5 (9.9, 11.0)
2001	245	11.8 (0.7)	11.7 (11.3, 12.3)
2002	173	11.8 (0.6)	11.9 (11.5, 12.2)
2003	106	11.7 (0.8)	11.7 (11.4, 12.2)
2004	169	10.8 (0.9)	10.8 (10.4, 11.3)
2005	193	10.8 (1.0)	11.0 (10.4, 11.4)
2006	126	10.2 (0.9)	10.2 (9.7, 10.8)
2007	116	9.3 (0.9)	9.3 (9.0, 9.8)
2008	147	10.1 (0.9)	10.2 (9.7, 10.7)
2009	104	9.0 (0.9)	9.0 (8.6, 9.5)
2010	93	8.0 (1.0)	8.0 (7.5, 8.5)
2011	102	8.4 (0.8)	8.5 (8.1, 9.0)
PM_2.5_, particulate matter ≤ 2.5 μm in diameter.			

**Figure 2:**
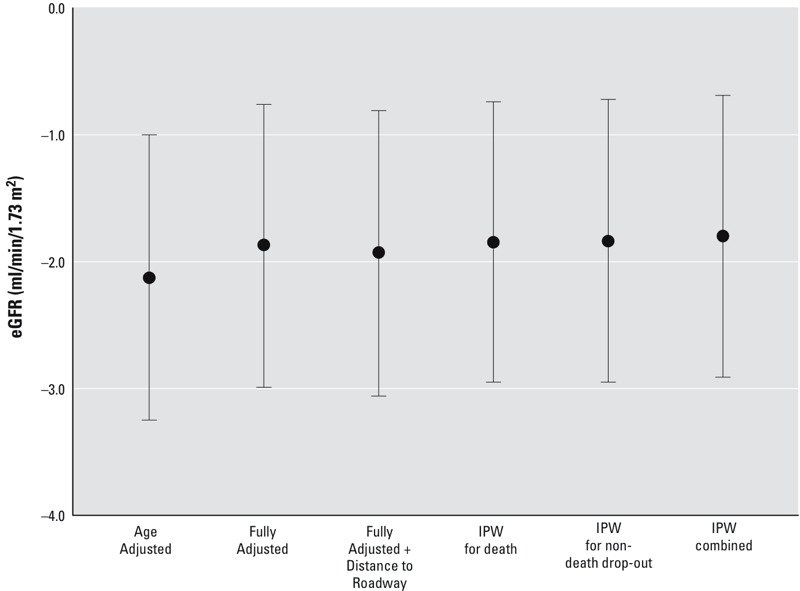
Adjusted differences in eGFR per 2.1 μg/m^3^ interquartile range increase in 1-year PM_2.5_. Associations were estimated in time-varying linear mixed-effect models of eGFR with random intercept for study participant. Age-adjusted models included adjustment for time since first visit and age at first visit. Fully adjusted models included additional adjustment for BMI, total cholesterol, diabetes, coronary heart disease, ARB medication, ACEI medication, other anti-hypertensive medication, years of education, percentage below poverty level in census tract, parental homeownership, smoking status, cumulative pack-years smoked, and daily alcohol intake. For the fully adjusted model, we also adjusted for distance to roadway and applied stabilized IPW for censoring by death and nondeath dropout and for the combination of both censoring mechanisms. Abbreviations: ACEI, angiotensin converting enzyme inhibitor; ARB, angiotensin receptor blocker; BMI, body mass index; eGFR, estimated glomerular filtration rate; IPW, inverse probability weight; PM_2.5_, fine particulate matter ≤ 2.5 μm in diameter.

The associations between 1-year PM_2.5_ and eGFR in all participants, after various adjustments, are summarized in Figure 2. One-year averaged exposure to PM_2.5_ was statistically significantly (*p* < 0.05) associated with lower eGFR in the overall sample. In the age-adjusted model, a 2.1-μg/m^3^ IQR higher 1-year PM_2.5_ was associated with a 2.13-mL/min/1.73 m^2^ lower eGFR [95% confidence interval (CI): –3.25, –0.76]. Relative to the age-adjusted model, the association between 1-year PM_2.5_ and eGFR in the fully adjusted model was slightly attenuated; a 2.1-μg/m^3^ IQR higher 1-year PM_2.5_ was associated with a 1.87-mL/min/1.73 m^2^ lower eGFR (95% CI: –2.99, –0.76) after adjustment for all other covariates. However, the association with the fully adjusted model was robust to additional adjustment for distance to roadway, and to inverse probability of censoring weights by death and nondeath dropout and by the combined weight of both censoring mechanisms.

**Figure 3:**
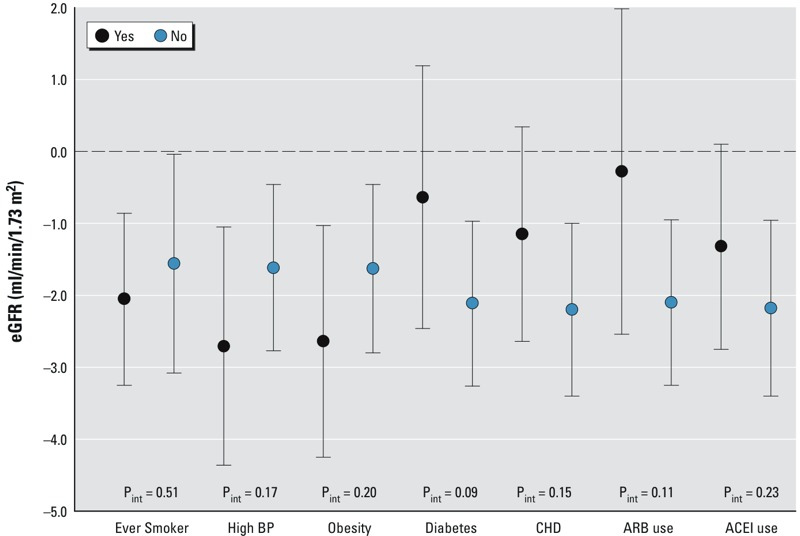
Adjusted differences in eGFR per 2.1 μg/m^3^ interquartile range increase in 1-year PM_2.5_ in subgroups according to participant characteristics. Associations were estimated in time-varying linear mixed-effect models of eGFR with random intercept for study participant after adjustment for time since first visit, age at first visit, BMI, total cholesterol, diabetes, coronary heart disease, ARB medication, ACEI medication, other anti-hypertensive medication, years of education, percentage below poverty level in census tract, parental homeownership, smoking status, cumulative pack-years smoked, and daily alcohol intake. Effect estimates presented for each subgroup were estimated from the nested interaction model. Abbreviations: ACEI, angiotensin converting enzyme inhibitor; ARB, angiotensin receptor blocker; BP, blood pressure; CHD, coronary heart disease; eGFR, estimated glomerular filtration rate; P_int_ = *p*-value for interaction; PM_2.5_, fine particulate matter ≤ 2.5 μm in diameter.

There was no statistically significant effect modification by participant characteristics on the association between 1-year PM_2.5_ and eGFR (Figure 3). Higher 1-year PM_2.5_ was at least marginally significantly (*p* < 0.10) associated with lower eGFR for the majority of subgroups, and the 95% confidence intervals of the effect estimates for the subgroups of each modifier widely overlapped each other. However, the negative association between 1-year PM_2.5_ and eGFR in the overall sample, as shown in Figure 2, was observed mainly for individuals not using ARB medication (β: –2.10 mL/min/1.73 m^2^, 95% CI: –3.25, –0.95); a null association was observed for individuals using ARB medication (β: –0.28 mL/min/1.73 m^2^, 95% CI: –2.54, –1.98). A similar pattern was observed for diabetes; the negative association between 1-year PM_2.5_ and eGFR was more pronounced for nondiabetic individuals (β: –2.11 mL/min/1.73 m^2^, 95% CI: –3.26, –0.97) than for diabetic individuals (β: –0.64 mL/min/1.73 m^2^, 95% CI: –2.46, –0.93) (*p*_interaction_ = 0.09). After additional adjustment for the 1-year PM_2.5_–ARB interaction in the model, there was less contrast in the 1-year PM_2.5_–eGFR association between diabetic (β: –1.00 mL/min/1.73 m^2^, 95% CI: –2.91, 0.90) and nondiabetic subgroups (β: –2.27 mL/min/1.73 m^2^, 95% CI: –3.44, 1.10) (*p*_interaction_ = 0.16). In contrast to the observations for diabetes, the negative associations between 1-year PM_2.5_ and eGFR were more pronounced for individuals presenting with obesity (β: –2.64 mL/min/1.73 m^2^, 95% CI: –4.25, –1.03) and with high BP (β: –2.71 mL/min/1.73 m^2^, 95% CI: –4.36, –1.05) than for individuals who did not present with obesity (β: –1.63 mL/min/1.73 m^2^, 95% CI: –2.80, –0.46) or with high BP (β: –1.62 mL/min/1.73 m^2^, 95% CI: –2.77, –0.46) (*p*_interaction_ = 0.20 and *p*_interaction_ = 0.17 for obesity and high BP, respectively).

**Figure 4:**
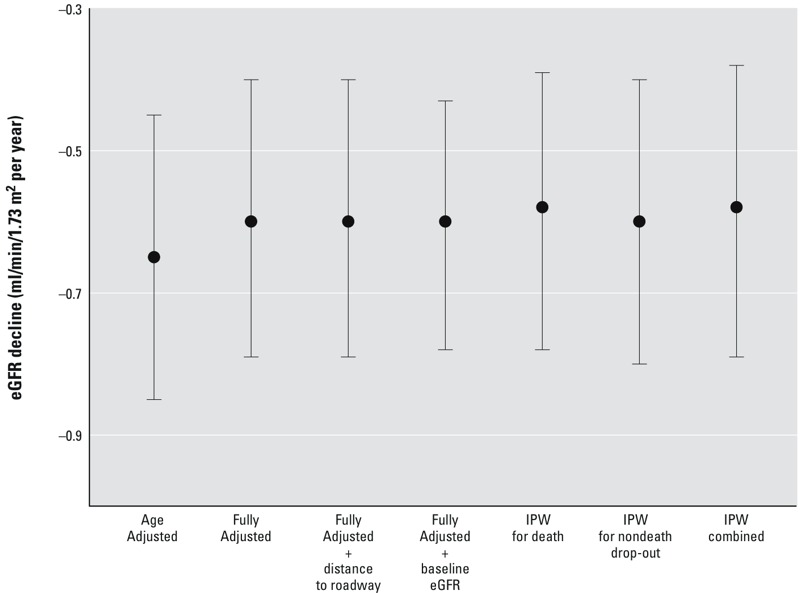
Adjusted difference in annual change in eGFR since first visit (mL/min/1.73 m^2^/year) per 2.1 μg/m^3^ interquartile range increase in 1-year PM_2.5_. Associations were estimated in time-varying linear mixed-effect models of eGFR with random intercept for study participant. Age-adjusted models included adjustment for time since first visit and age at first visit. Fully adjusted models included additional adjustment for BMI, total cholesterol, diabetes, coronary heart disease, ARB medication, ACEI medication, other anti-hypertensive medication, years of education, percentage below poverty level in census tract, parental homeownership, smoking status, cumulative pack-years smoked, and daily alcohol intake. For the fully adjusted model, we also adjusted for distance to roadway and baseline eGFR and applied stabilized IPW for censoring by death and nondeath dropout and for the combination of both censoring mechanisms. The effect estimates presented for each model were estimated from the interaction between time since first visit and 1-year PM_2.5_. Abbreviations: ACEI, angiotensin converting enzyme inhibitor; ARB, angiotensin receptor blocker; BMI, body mass index; eGFR, estimated glomerular filtration rate; IPW, inverse probability weight; PM_2.5_, fine particulate matter ≤ 2.5 μm in diameter.

One-year PM_2.5_ was associated with a higher rate of decline in eGFR over time; an interaction was identified between time since first visit and 1-year PM_2.5_ (*p*_interaction_ < 0.0001) (Figure 4). After adjustment for all covariates, a 2.1-μg/m^3^ higher 1-year PM_2.5_ was associated with an additional annual decrease in eGFR of 0.60 mL/min/1.73 m^2^/year (95% CI: –0.79, –0.40). This association was robust to additional adjustments for distance to roadway and for eGFR measured at the first visit, and to inverse probability of censoring weights by death and nondeath dropout and by the combined weight of both censoring mechanisms.

### Secondary Analyses

Similar findings were observed for the association between 1-year PM_2.5_ and eGFR from the baseline cross-sectional analysis; in the fully adjusted model, a 2.1-μg/m^3^ higher 1-year PM_2.5_ was associated with a 1.63-mL/min/1.73 m^2^ lower eGFR (95% CI: –3.78, 0.51). No significant effect modification (*p*_interaction_ = 0.27) was observed for impaired renal function at the first visit on the association between 1-year PM_2.5_ and eGFR; negative associations between 1-year PM_2.5_ and eGFR were observed for individuals who began the study period with (β: –2.40 mL/min/1.73 m^2^, 95% CI: –4.11, –0.61) and without (β: –1.41 mL/min/1.73 m^2^, 95% CI: –2.47, –0.35) impaired renal function. Similarly, age-related decline in eGFR associated with 1-year PM_2.5_ was observed for individuals who began the study period with (β: –0.53 mL/min/1.73 m^2^/year, 95% CI: –0.74, –0.33) and without (β: –0.65 mL/min/1.73 m^2^/year, 95% CI: –0.84, –0.46) impaired renal function. For the mediation analysis, we observed that a 2.1 μg/m^3^ increase in 1-year PM_2.5_ was associated with an estimated 0.14-mL/min/1.73 m^2^ lower eGFR (95% CI: –0.28, –0.01) through a 1-mmHg increase in DBP, assuming that the identification assumptions were met and that there was no time-varying confounding with respect to 1-year PM_2.5_ and DBP.

## Discussion

The novel findings from this prospective cohort study of older men indicate that 1-year PM_2.5_ exposure is associated with reduced eGFR and an increased rate of eGFR decline over time. These associations were robust to various sensitivity analyses. There was no statistically significant effect modification by participant characteristics on the association between 1-year PM_2.5_ and eGFR, but the associations were null for ARB users and for diabetics. The association between 1-year PM_2.5_ and eGFR was also mediated by an increase in DBP, although this finding is based on some unverifiable assumptions.

To date, there has been limited research on the relationship between long-term exposure to particulate matter air pollution and renal-related outcomes. A recent cross-sectional study of 1,103 stroke patients in the Boston metropolitan area found that participants living < 50 m from a major roadway had a 3.9-mL/min/1.73 m^2^ lower eGFR (95% CI: –1.0, –6.7) than participants who lived >1,000 m away (Lue et al. 2013). An earlier longitudinal analysis of 3,901 participants within the Multi-Ethnic Study of Atherosclerosis reported that chronic exposures to PM_2.5_ and PM_10_, which were averaged over 20 years, were not associated with the urinary albumin/creatinine ratio, although there was an elevated risk of microalbuminuria associated with chronic PM_10_ exposure that was of marginal statistical significance (O’Neill et al. 2008).

Although we did not identify a statistically significant interaction between 1-year PM_2.5_ and ARB use, it is of interest that the association between 1-year PM_2.5_ and eGFR was null for ARB users. ARBs, alone or in combination with ACEIs, have been shown to slow the progression of chronic kidney disease in individuals with or without diabetes, independent of their blood pressure–lowering effect (Palmer et al. 2015). Findings from experimental *in vivo* and *in vitro* studies also suggest that ARBs may minimize the oxidative stress and vascoconstrictive effects of PM (Ghelfi et al. 2010; Li et al. 2005). Here, we observed that the negative association between 1-year PM_2.5_ and eGFR was stronger for obese participants but weaker for diabetic participants, which was unexpected. The pattern of effect modification by diabetes was similar to that of ARB use, and in this study, the prevalence of ARB use was two times greater in diabetic (36%) than in nondiabetic participants (18%).

The underlying biological mechanism(s) that may explain the novel association between long-term PM_2.5_ exposure and lower eGFR and higher eGFR decline are not known. We hypothesized that the negative association between 1-year PM_2.5_ exposure and eGFR was mediated by an increase in DBP, and the result from the mediation analysis appears consistent with that hypothesis. If the assumptions of the causal mediation analysis hold, the small magnitude of the estimated mediated effect for PM_2.5_ also suggests that the association between 1-year PM_2.5_ and eGFR is likely only partially mediated by DBP and that there may be nonhemodynamic pathways to consider. Previous studies have revealed associations between long-term PM_2.5_ exposures and vascular/endothelial dysfunction (Hoffmann et al. 2007; Krishnan et al. 2012; Wilker et al. 2014). In addition, observational evidence supports the associations between markers of vascular/endothelial dysfunction and serum creatinine-derived eGFR decline (Foster et al. 2013; Perticone et al. 2010). Taking these findings together, we also hypothesize that the associations between 1-year PM_2.5_ and both eGFR and eGFR decline are possibly mediated by pathways associated with vascular/endothelial dysfunction.

The clinical relevance of the present findings merits further discussion. The difference in eGFR for a 2.1-μg/m^3^ increase in 1-year PM_2.5_ is comparable in magnitude to the reduction in eGFR observed for a 2-year increase in age in this cohort (β: –1.8 mL/min/1.73 m^2^, 95% CI: –2.1, –1.5). In the context of eGFR decline, a rapid decline in creatinine-based eGFR of ≥ 3 mL/min/1.73 m^2^ per year in older adults has been shown to be associated with a 70% higher risk of cardiovascular mortality (Rifkin et al. 2008). Extrapolating our findings for a 10-μg/m^3^ increase, which may be more appropriate in more polluted settings, may result in a more clinically meaningful estimate of association for eGFR decline, particularly in older populations.

This study has a number of strengths, including a prospective design to investigate the role of PM_2.5_ exposure on repeated measures of serum creatinine–derived eGFR, the use of validated spatiotemporal models for assessment of PM_2.5_, advanced methods to address potential bias from age-dependent censoring by death and nondeath dropout, and adjustment for multiple potential confounders and individual-level risk factors. However, there are limitations to consider in this study. Although the CKD-EPI equation for estimating eGFR has shown clinical utility in risk prediction of end-stage renal disease and cardiovascular outcomes (Matsushita et al. 2010), there is an inherent physiologic limitation to using serum creatinine as a marker of kidney filtration (Shemesh et al. 1985). Non-GFR determinants of eGFR, including muscle mass and diet, may lead to overestimation of serum creatinine, particularly for individuals with preserved renal function, and future studies should consider using alternative agents for estimation of eGFR including cystatin C (Fan et al. 2014). We hypothesize that any error in measurement of eGFR is likely independent of the 1-year PM_2.5_ predictions assigned to the participants in the present study and would result in bias of the true association between 1-year PM_2.5_ and eGFR toward the null. The potential for reverse causation, particularly between DBP and eGFR, is not theoretical and is also a limitation of our mediation findings; there is limited observational evidence suggesting that reduced eGFR may precede elevation in blood pressure and onset of hypertension (Kestenbaum et al. 2008; Takase et al. 2012). Potential risk factors or confounding variables included in our models, such as diabetes status and BMI, may also be intermediate variables. Additionally, there may be some unmeasured confounding from other factors associated with eGFR that may be correlated with PM_2.5_ exposure in this study, such as environmental noise. Traffic, aircraft, and railway noise has been shown to be associated with markers of cardiovascular disease (Münzel et al. 2014), but whether environmental noise is associated with renal function is unknown. However, our association persisted after controlling for distance to major roadway, which is a marker for traffic-related pollution including noise. Finally, this study consists of older men who are predominantly White and reside in a lightly polluted area. The observed findings may not be generalizable to women, younger individuals, other racial and ethnic groups, or people living in other areas owing to differential environmental and physiological factors.

## Conclusion

In summary, we found that long-term PM_2.5_ exposure was associated with reduced eGFR and increased age-related eGFR decline in this longitudinal sample of predominantly older White men. These findings support the hypothesis that long-term PM_2.5_ exposure negatively affects renal function and renal function decline. These findings should be verified in other study populations with longitudinal follow-up.
